# NLRP12-associated systemic autoinflammatory diseases in children

**DOI:** 10.1186/s12969-022-00669-8

**Published:** 2022-02-05

**Authors:** Hui-fang Wang

**Affiliations:** Department of Pediatrics, The First Affiliated Hospital of Zhengzhou University, 1# Jianshe east Rd, Zhengzhou, 450052 Henan China

**Keywords:** NLRP12, Systemic autoinflammatory diseases, Recurrent fever, Multisystem inflammation, IL-1-related inflammatory pathways

## Abstract

Systemic autoinflammatory diseases (SAIDs) are a group of monogenic diseases characterized by disordered innate immunity, which causes excessive activation of inflammatory pathways. Nucleotide-binding leucine-rich repeat-containing receptor 12-related autoinflammatory disease (NLRP12-AID) is a newly identified SAID and a rare autosomal dominant disorder caused by mutations in the NLRP12 gene, which is also known as familial cold autoinflammatory syndrome 2 (FCAS2) and mostly occurs in childhood. A total of 33 cases of NLRP12-AID in children and 21 different mutation types have been reported to date. The disease is mainly characterized by periodic fever, accompanied by multisystem inflammatory damage. NLRP12-AID is diagnosed through early clinical identification and genetic detection. Emerging drugs targeting interleukin-1-related inflammatory pathways are expected to change the treatment options and improve the quality of life of pediatric patients. This article aims to summarize the characteristics and pathogenesis of reported NLRP12-AID cases in children and provide ideas for clinical diagnosis and treatment.

## Background

Systemic autoinflammatory diseases (SAIDs) are a group of rare monogenic diseases. In contrast to autoimmune diseases, SAIDs are characterized by excessive production of proinflammatory cytokines caused by an imbalance in the innate immune system, with clinical manifestations including recurrent fever, a multisystem inflammatory response, and immunodeficiency [1–4]. In 1997, the French Familial Mediterranean Fever (FMF) Consortium [5, 6] first reported a monogenic disease characterized by fever and multisystem inflammation, i.e., FMF caused by MEFV mutations. In 1999, McDermott et al. [7] reported that TNFRSF1 mutations cause tumor necrosis factor (TNF) receptor-associated periodic syndrome (TRAPS) and first proposed the term “autoinflammation”. Since then, with the popularization of genome sequences, especially whole-exome sequencing technology in clinical practice, the spectrum of SAIDs has rapidly and continuously expanded. MVK mutation-induced hyperimmunoglobulin D syndrome (HIDS) [8, 9] and NLRP3 mutation-induced cryopyrin-associated periodic syndrome (CAPS) [10–13] have been added to the spectrum of SAIDs. To date, more than 30 SAIDs have been reported and these disorders are related to excessive inflammasome activation, elevated reactive oxygen species, autophagy disorders, and other pathological mechanisms [14].

The nucleotide-binding leucine-rich repeat-containing receptor 12 (NLRP12) gene is another member of the NLRP family and an innate immune sensor. It is activated by foreign microbial proteins and damaged intracellular components to regulate downstream inflammatory pathways and plays an important role in steady-state regulation of innate immunity [15, 16]. Recent studies have demonstrated that NLRP12 mutations can cause a rare autosomal dominant SAID (NLRP12-AID) known as familial cold autoinflammatory syndrome 2 (FCAS2), which is mostly induced by cold [17, 18]. The molecular mechanism involves excessive release of inflammatory cytokines, such as interleukin (IL)-1β, caused by weakened inhibition of the nuclear factor kappa B (NF-κB) pathway due to the decreased functional activity of mutated NLRP12 [17–20]. Because of the genetic mutation, most cases have an early onset in childhood. This disease is relatively difficult to diagnose because of its rarity but seriously affects the physical and mental health and quality of life of pediatric patients. In order to provide pediatricians some references for early diagnosis and treatment, we review reported pediatric cases and discuss NLRP12-AID pathogenesis, clinical characteristics, and emerging treatment protocols targeting IL-1-related inflammatory pathways in this article.

### Pathogenesis of NLRP12-associated autoinflammatory disease

Nucleotide-binding domain and leucine-rich repeat-containing receptors (NLRs), also known as Nod-like receptors, are a group of highly conserved cytoplasmic pattern recognition receptors. They play important roles in initiating the innate immune response [21, 22]. Their common structural features are a leucine-rich repeat (LRR) domain at the C-terminus; the central NACHT domain and the N-terminus, which contains a specific effector domain that can mediate the interaction between homologous proteins and initiate downstream signaling pathways [15, 16, 23]. NLRs are divided into 4 major families based on different N-terminal effector domains: NLRP, NLRA, NLRB and NLRC/X. The pyrin domain (PYD) is a unique N-terminal effector domain of NLRP subfamilies [23]. It has been shown that NLRP12 can exert both anti-inflammatory and antitumor effects by downregulating the production of various proinflammatory cytokines and chemokines by inhibiting the classical and nonclassical NF-κB activation pathways and the mitogen-activated protein kinase (MAPK)/extracellular signal-regulated kinase (ERK) signaling pathway [24, 25]. During infection by specific pathogens, such as *Yersinia pestis* and *Plasmodium*, NLRP12 can drive the activation of caspase-1 as an important component of the inflammasome, resulting in the lysis of IL-1β and IL-18 precursors into mature forms to resist pathogens [26]. In addition, NLRP12 also plays a key role in the adaptive immune response by regulating the migration of T cells, neutrophils and dendritic cells and the expression of major histocompatibility complex (MHC) class I molecules [27].

**Fig. 1:**
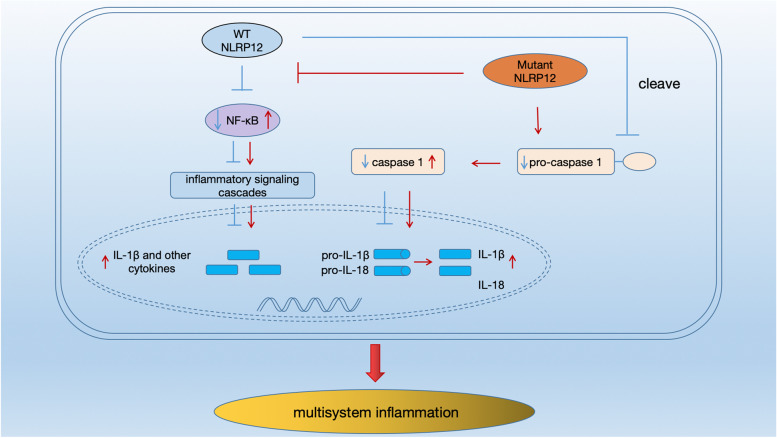
Explanatory diagram of signaling pathways involved in NLRP12-AID. Mutant NLRP12 disrupts its inhibitory effect on the NF-κB pathway and enhances caspase-1 activation, resulting in the excessive production of IL-1β and other inflammatory cytokines, which are involved in multisystem inflammation in NLRP12-AID

Recent results suggested that a mutated functional domain in NLRP12 not only diminishes the inhibitory effect on NF-κB but also enhances caspase-1 activity, which eventually leads to excessive production of various inflammatory cytokines, such as IL-1β [17–19, 24] (Fig. 1). IL-1β, which is an “alarm cytokine”, has a powerful proinflammatory effect in innate immunity [13], which can stimulate the production of other inflammatory cytokines (such as IL-6 and TNF-α) and also self-induce to further amplify the inflammatory response [12, 13, 28]. As a powerful endogenous pyrogen, IL-1β can increase body temperature and cooperate with other inflammatory cytokines (such as TNF, inducible nitric oxide synthase (iNOS), cyclooxygenase (COX) 2, and prostaglandin E2 (PGE2)) to cause inflammatory responses in multiple systems, which is associated with recurrent fever, multisystem inflammatory syndrome, and elevated acute-phase reactive proteins during the onset of SAIDs [12, 13]. Borghini et al. [20] reported a rapid increase in IL-1β and reactive oxygen species in patients with NLRP12-AID. Jéru et al. [11] found that that intracellular IL-1β and IL-6 levels in children with NLRP12-AID were 80–175 times and 43–110 times higher than those in control children, respectively. After further stimulating peripheral blood mononuclear cells (PBMCs) with lipopolysaccharides, the IL-1β level of pediatric NLRP12-AID patients was still 5–10 times that in the control group, and similar results were observed for IL-1Ra, IL-6, and TNF. Administration of the exogenous IL-1 receptor antagonist anakinra, the IL-1β level decreased significantly and clinical symptoms quickly resolved, demonstrating that an imbalanced inflammatory response had occurred. Therefore, uncontrolled IL-1β release is an important pathological basis for NLRP12-AID, and exogenous IL-1 related inhibitors can antagonize the excessive activation of inflammatory pathways and return them to a steady state [12, 13, 28, 29].

### Clinical characteristics of NLRP12-AID

All relevant literature in PubMed was searched from the database’s establishment to November 2020. Jéru et al. [17] first reported autoinflammatory diseases related to NLRP12 mutations in 2008. Approximately 80 related cases have been reported worldwide [30–32], including only 33 pediatric cases. To summarize the characteristics of pediatric cases and provide evidence for early diagnosis by pediatricians, we analyzed the clinical characteristics of the 33 cases of NLRP12-AID diagnosed in childhood (Table 1). The age at diagnosis ranged from 2 months to 17 years old. Among the children whose sex was known, 11 (35%) were male, and 20 (65%) were female. Four patients (22%) had a positive family history, and 10 cases (30%) were clearly induced by cold stimulation. The most common clinical symptom was periodic fever lasting for different durations (100%). Other symptoms included polyarthralgia/arthritis in 18 patients (55%), abdominal pain/diarrhea in 16 patients (48%), rash in 15 patients (45%) (urticaria in 11 patients), lymphadenopathy/splenomegaly in 11 patients (33%), headache in 8 patients (24%), neurosensory deafness in 7 patients (21%), aphthous stomatitis in 4 patients (12%), and elevated acute-phase reactants such as C-reactive protein (CRP)/erythrocyte sedimentation rate (ESR) in 18 patients (55%). Some of these patients had comorbid diseases caused by acquired immune disorders, including Crohn’s disease, C3 glomerulopathy, juvenile idiopathic arthritis, autoimmune hemolytic anemia, and susceptibility to infection. Disruptions in innate immune signaling molecules, such as IL-1β/IL-1R, have also been demonstrated to play important roles in the activation and maintenance of adaptive immune pathways [12, 40].

**Table 1:** Comparison of the clinical manifestations of 33 pediatric patients with NLRP12-AID summarized in the literature

Publication year [ref]	age/sex	NLRP12 variant	Family history	Cold trigger	Fever duration	Interval	Polyarthralgia/arthritis	Abdominal symptoms	Skin rash	Lympadenopathy/splenomegaly	Headache	Neurosensory deafness	Other
2008 [17]	10/M (twins)	c.850C > T; p.R284X	+	+	2–10 d	< 1 m	+	–	Urticaria	–	+	+	
			+	+	2–10 d	< 1 m	+	–	Urticaria	–	+	+	
	9/F	c.2072 + 3insT; p.V635fs	+	+	7 d	3 w	+	+	–	+	+	–	Buccal aphthous ulcers; Increased CRP
2011 [18]	1/?	c.1054C > T; p.R352C	–	+	2–3 d	< 1 m	+	+	–	–	–	–	
	2.5/?		–	+	< 1 d	> 1 w	+	+	Malar rash	+	–	–	Buccal aphthosis; increased CRP
2013 [33]	3/F	c.1206C > G; p.F402L	+	+	3d		+	–	Urticarial	+	–	–	Increased ESR
	17/F		–	+	5-10d		+	–	maculopapular and urticarial	–	+	+	Increased CRP/ESR
2017 [34]	2 m-13 y (9/F, 6/M)	**10/15 single heterozygous mutations:** c.154G > A; p.G52S c.169G > T; p.A57S c.632G > A;p.R211H c.737A > G; p.Y246C c.1054C > T; p.R352C c.1250A > T;p.Q417L c.2216_2217del;p.R739fs c.910C > T; p.H304Y **3/15 heterozygous:** c.910C > T; p.H304Y&c.1206C > G; p.F402L(2) c.2168G > A; p.R723Q &c.2261G > A; p.R754H(1) **2/15 homozygous:** c.1206C > G; p.F402L(2)	?	3/5	1–5 d	1 w–2-3 m	3/15	8/15	2/15 Urticaria	4/15	3/15	–	9/15 Infection susceptibility, 3/15 Crohn’s disease, 2/15 aphthous stomatitis, 1/15 immunodeficiency, autoimmune hemolytic anemia, pancytopenia and interstitial lung disease; 3/15 Increased CRP
2018 [35]	6/M	c.1732A > G;p.S578G	–	–	4–5 d	1 m	–	+	Urticaria	–	+	–	C3 glomerulopathy, increased CRP/ESR
2019 [36]	9/M	c.779C > T; p.T260M	–	–	3–4 d	4–6 w	–	+	–	–	–	–	Increased CRP
2019 [37]	4/F	c.2935A > G; p.S979G	–	–	14 d	1–2 m	+	+	–	+	–	+	Neutrophilic leucocytosis, thrombocytosis and increased acute phase reactants
2020 [38]	3/F	c.1742G > A; p.W581X	–	–	+		+	–	Macular rash	–	–	–	Increased CRP/ESR, ANA+
	5/F	c.2072 + 2dupT	–	–	+		+	–	Urticaria	+	–	+	Increased CRP/ESR
	0.5/F	c.1673 T > G; p.L558R	–	–	2–8 d	1–4 m	+	+	Rash	–	–	–	Hearing loss and uveitis, increased ESR/CRP/platelets
2020 [32]	4/(3/F, 1/M)	c.1673 T > G; p.L558R	–	–	4/4		3/4	1/4	3/4 Urticaria	2/4	–	2/4	4/4 Increased ESR/CRP, 1/4 ANA+
		c.1742G > A; p.W581X											
		c.2188G > C; p.G730R											
		c.2072 + 2dupT											
2020 [39]	9/F	c.2129 T > C, p.L710P	–	–	10 d	3 m	+	–	Rash	–	–	–	Increased CRP/ESR/IL-6 serum ferritin
Summary	33cases	21 variants	4/18	10/33	33/33		18/33	16/33	15/33	11/33	8/33	7/33	18/33 Increased acute phase reactants; 4/33 aphthosis

*M* male, *F* female, *d* days, *m* months, *y* years, *CRP* C-reactive protein, *ESR* erythrocyte sedimentation rate, *ANA* anti-nuclear antibodies

**Fig. 2:**
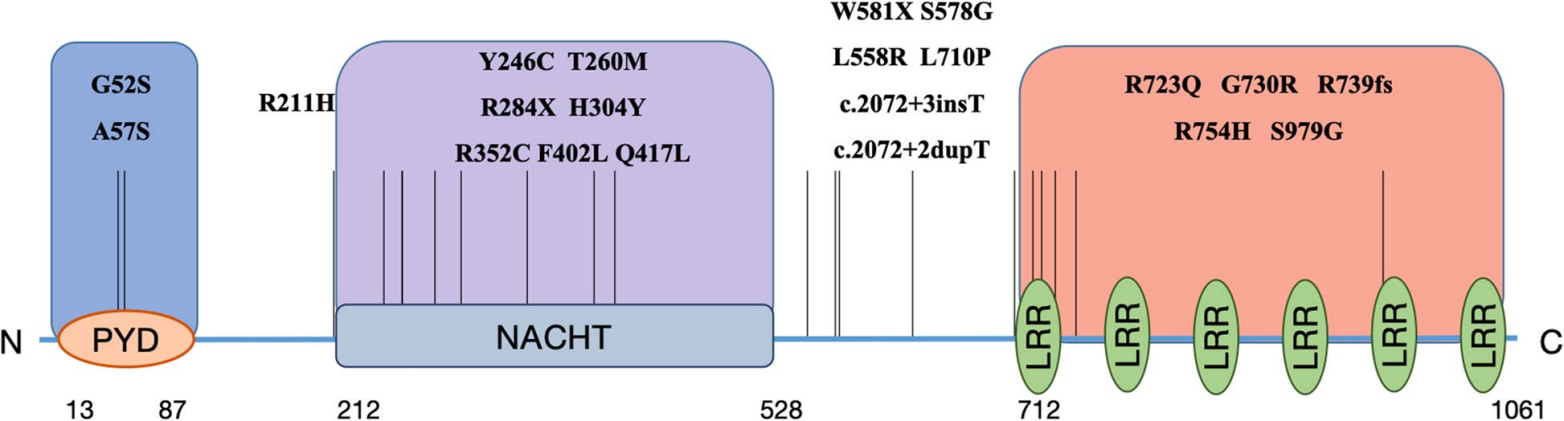
Schematic outline of mutations in the domains of NLRP12. Schematic diagram of reported mutation sites in different domains of NLRP12, including 2 mutation sites in the PYD domain, 7 mutation sites in the NACHT domains, and 5mutation sites in the LRR domain

To date, 21 different types of NLRP12 mutations have been discovered, including 16 missense mutations (76%), 2 nonsense mutations (10%), and 3 frameshift mutations (14%). Most mutation sites are located in its functional domains (Fig. 2), which may impair the anti-inflammatory function of NLRP12, leading to a subverted pattern of inflammation [41]. The most reported mutation is c.1206C > G; p.F402L. Six cases of this site mutation were noted in our statistical data, 2 of which involved homozygous mutations, but p.F402L also has a higher occurrence rate in the normal population (3 to 5%) [34]; thus, its pathogenicity is still controversial. Notably, NLRP12 mutations have also been detected in the first-degree relatives in many cases, but no corresponding suspicious clinical symptoms were reported, indicating that NLRP12 mutations combined with other genetic and environmental factors may be the root cause of this disease.

### Diagnosis of NLRP12-AID

The most important clinical symptom of NLRP12-AID is noninfectious recurrent fever. Fever may exhibit a certain pattern, lasting 2–10 days each time, and has self-limiting characteristics with no special manifestations during onset [42, 43]. Torreggiani et al. [44] defined periodic or recurrent fever as 3 or more unexplainable fever episodes within 6 months, with an interval of at least 7 days between 2 fever episodes. Statler et al. [45] reported that unexplainable fevers accounted for as many as 69% of fever cases in children, complicating determination of the cause of fever, especially recurrent fever. After excluding infectious and neoplastic factors, autoinflammatory diseases caused by immune system disorders should be considered. The onset of most NLRP12-AID symptoms is related to cold stimulation [17]. Due to its dominant genetic background, a positive family history is critical information for diagnosing NLRP12-AID, but some family members who carry mutated genes have no obvious clinical symptoms due to environmental factors or new mutations [43]. For most SAIDs, the initial onset occurs during childhood, even before the age of 10 years; therefore, the age at initial onset should be traced for patients with a delayed diagnosis [43]. Due to multisystem inflammatory responses, accompanying symptoms of NLRP12-AID can include polyarthralgia/arthritis, abdominal pain/diarrhea, lymphadenopathy/splenomegaly, headache, neurosensory deafness, aphthous stomatitis and so on. Rashes (urticaria, erythema and papules) related to fever and systemic inflammatory responses are also characteristic manifestations, that are significantly associated with cold stimulation, with pigmentation loss after the rashes subside [17, 20]. Generally, the interval between onset is not completely fixed, but the intermittent normalized clinical symptoms and laboratory indicators can distinguish SAIDs from other chronic diseases [46]. The response of SAIDs to antibiotics and immunosuppressive therapy is usually poor, and corticosteroids can relieve certain symptoms in some cases.

Gattorno et al. [47] established a diagnostic scoring system for some SAIDs; which is used to calculate scores based on 6 variables (young age at onset, positive family history of periodic fever, thoracic pain, abdominal pain, diarrhea and oral aphthosis) and divide individuals into high-risk and low-risk groups. Therefore, we emphasize that family history, onset patterns and treatment responses must be fully considered in for pediatric patients with recurrent fever, and these data and targeted clinical examination results must be systematically summarized and analyzed [43]. Clinicians should conduct timely genetic analyses for highly suspected patients based on medical history. Eventually, the genetic test results must be analyzed in conjunction with the patients’ clinical symptoms. Early diagnosis and treatment can not only improve their quality of life but also prevent irreversible long-term complications.

### Treatment protocols for NLRP12-AID

The goal of SAID treatment is to suppress the persistent hyperinflammatory state, restore the function of multiple systems and improve the quality of life of patients. Because the pathological mechanism is still unclear, few treatment protocols are available. Many reports suggest that colchicine is ineffective [17, 36], but some cases respond well to corticosteroids [33, 48]. Although oral corticosteroids can shorten the fever duration or reduce fever intensity, they cannot completely control the disease [17, 34], and symptoms often recur after reducing the dosage [39]. Long-term use of corticosteroids has some potential adverse effects, such as the induction or aggravation of infection, Cushing syndrome, stress ulcers, growth inhibition hypertension, and metabolic effects (Table 2).

**Table 2:** Summary of the therapeutic effects of different protocals for NLRP12-AID

Drug	Age/Sex	Dosage or administration	Curative effect	References
Colchicine	10/M (twins)	NK	↓ Fever(≈38 °C) but NE on the frequency of episodes	[17]
	9/F	NK	NE	
	9/M	NK	NE	[36]
Corticosteroids	9/F	Oral administration	↓Length of febrile episodes(3 ~ 5d)	[17]
	2 m-13y/F	Short courses(1-3d)	Shorter and milder fever episodes but not CR	[34]
	9/F	Prednisolone (1.5 mg/kg by mouth daily) and methotrexate (10 mg/m2 by mouth weekly)	Inflammatory parameters are still abnormal, but without any symptoms	[39]
	3/F	prednisolone(dose NK)	CR	[33]
	17/F	prednisolone(up to 50 mg/daily)	CR	[33]
	4/F	Prednisolone	Good response	[37]
NSAIDs	3/F	NK	PR	[33]
	17/F	NK	PR	[33]
NSAIDs+Corticosteroids	9/M	Naproxen+prednisolone(15 mg/day×7d, and once when paroxysms started)	PR	[36]
Anakinra	17/F	100 mg/daily	CR	[33]
	6/M	1 mg/kg/day	CR	[35]
	10/M(twins)	1 mg/kg/day	CR in 2 months but relapse in 14 months	[11]
Canakinumab	2 m-13y/F	From 2 mg/kg every 8 weeks to 5 mg/kg every 4 weeks	Resolution of the rash, abdominal pain and arthralgia; fever episodes became rare and very mild	[34]
	6/M	2 mg/kg/month	CR	[35]
TNF inhibitors (adalimumab and infliximab)	2 m-13 y/M	NK	Shorter and milder fever episodes but not CR	[34]

*M* male, *F* female, *NK* not known, *NE* no effect, *CR* complete remission, *PR* partial remission

Because excessive IL-1β production initiates the downstream inflammatory cascade and leads to multisystem tissue damage, drugs targeting IL-1-related inflammatory pathways may provide breakthroughs for treatment strategies. At present, 4 targeted drugs can block the IL-1 pathway [48]; among these drugs, anakinra is a short-acting human recombinant IL-1R antagonist that can competitively inhibit the binding of IL-lβ and IL-α to IL-1R and block IL-1 signal transduction. The Food and Drug Administration (FDA) approved anakinra for the treatment of rheumatoid arthritis and neonatal-onset multisystem inflammatory disease (NOMID) in pediatric patients older than 4 years, and the European Union approved anakinra for the treatment of rheumatoid arthritis, Still’s disease, and periodic fever syndrome (including all types of CAPS and FMF) in pediatric patients older than 8 months [49]. The National Institutes of Health conducted a prospective single-center clinical cohort study from 2003 to 2010 and found that anakinra has the same safety and tolerability in children and adults based on a 5-year follow-up survey of 43 CAPS patients [50]. The biological half-life is 4–6 h, and daily subcutaneous administration is required [51]. Vitale et al. [33] reported a case of a 17-year-old female pediatric patient with NLRP12-AID who received anakinra (100 mg/d), and her condition was quickly and continuously controlled. In another experimental study, Jéru [11] reported that a marked clinical improvement within 2 weeks of anakinra treatment (1 mg/kg/d) in 2 brothers with NLRP12-AID. A follow-up visit at 2 months revealed that their fevers were significantly relieved, skin and musculoskeletal manifestations had disappeared, and intracellular IL-1β levels were basically reduced to normal, but IL-1Ra, IL-6, and TNF levels remained elevated. During months 3–8 of treatment, each of the patients experienced 2 new episodes of moderate fever and systemic inflammation, and even developed severe disabling myalgia. Over the subsequent 6 months (treatments 9–14), the efficacy of anakinra was further reduced. Fever, arthralgia, and skin manifestations reappeared, and the intracellular levels of IL-1β, IL-1Ra, IL-6, and TNF also increased. Considering the biologically confirmed resistance to anti-IL-1therapy, anakinra treatment was terminated for these 2 patients. The mechanism of anakinra resistance has not been determined, but the cytokine network in the body is a complex regulatory system composed of synergistic and antagonistic mechanisms. Elevation of TNF and other inflammatory cytokines may be a potential mechanism [11].

Canakinumab is a long-acting humanized anti-monoclonal antibody that specifically binds to IL-1β and blocks it from binding to the IL-1 receptor. The FDA approved it for the treatment of periodic fever syndrome (including FMF, CAPS, HIDS/mevalonate kinase deficiency (MKD), and TRAPS) in adults and children over 2 years old. The European Union (EU) also approved canakinumab for the treatment of Still’s disease and gouty arthritis [49]. The half-life is approximately 21–28 days, and the drug can be injected once every 8 weeks. The initial doses of canakinumab are 4 mg/kg for 2-to-4-year-old children with body weight ≥ 7.5 kg and for children older than 4 years with body weight between 7.5 and 15 kg and 2 mg/kg for those weighing 15–40 kg; 150 mg can be administered directly to children weighing over 40 kg. The maintenance dose depends on the clinical response of each patient [52]. Kostik et al. [34] reported a case of a female NLRP12-AID patient with a severe clinical course (monthly periodic fever, splenomegaly, arthralgia, abdominal pain, episodic urticarial-like rash, growth delay, and high CRP) who was treated with canakinumab due to failed corticosteroids treatment. The initial dose of canakinumab was 2 mg/kg every 8 weeks which was gradually increased to 5 mg/kg every 4 weeks. This treatment led to resolution of her rash, abdominal pain, and arthralgia, and fever episodes became rare and very mild. Basaran et al. [35] reported a case of a male child with NLRP12-AID with C3 glomerulopathy who relapsed after symptom relief with glucocorticoids and cyclophosphamide. His skin rash disappeared within 24 h after anakinra (1 mg/kg/d) was first administered, and other symptoms were also ameliorated. CRP/ESR decreased to normal levels within 15 days, and bilateral papilledema resolved after 1 month. Because the frequent injections caused local pain and injection reactions, the treatment plan was adjusted to canakinumab 2 mg/kg once per month, and no relapse was observed at the 1-year follow-up.

Anakinra must be administered subcutaneously every day due to its short half-life, and local adverse reactions are common, limiting its application in some populations. Canakinumab can be used for patients who cannot tolerate anakinra, and the administration interval can be as long as 1 to 2 months. Compared with traditional therapies, IL-1 inhibitors have clear targets, cause fewer adverse reactions, and have much better efficacy. This drug is expected to improve the clinical prognosis of pediatric NLRP12-AID patients.

## Conclusions

SAIDs are a spectrum of innate immune disorders that induce multisystem inflammatory damage through excessive activation of inflammatory pathways, which lack specific antigens, high titers of autoantibodies, and abnormal activation of specific T-cell clones and human leukocyte antigen (HLA) class II genes, and the onset usually occurs at a young age. NLRP12-AID, a newly identified SAID, is inherited in an autosomal dominant manner. However, due to the rare genetic characteristics of such diseases, missed diagnosis or misdiagnosis often occurs. Currently, only a small number of relevant case reports are available. Here, we reviewed the data of 33 pediatric patients. The characteristic clinical symptoms are noninfectious periodic fevers accompanied by inflammatory symptoms in multiple systems such as the joints, muscles, digestive organs, skin, and nerves. Although the specific pathogenic mechanism of NLRP12 with different mutation types still requires further investigation, excessive production of IL-1β and other inflammatory cytokines due to imbalanced regulation of the innate immune system by NLRP12 undoubtedly plays an important role in the pathogenic process of NLRP12-AID. In addition, Crohn’s disease, C3 glomerulopathy, juvenile idiopathic arthritis and other autoimmune diseases in some patients are also closely related to the functional imbalance of NLRP12 in acquired immunity. Certainly, the cross-interactions between pathogenic NLRP12 mutations in autoinflammatory and autoimmune disease certainly still require further study. These clinical manifestations may also be the combined effects of NLRP12 mutations and other genetic and environmental factors.

No unified diagnosis or treatment protocol is available for NLRP12-AID. Patients with noninfectious recurrent fever and other immune or inflammation-related clinical manifestations should be alert to autoinflammatory diseases, and a complete genetic examination is recommended. In terms of treatment, monotherapy or the combined use of corticosteroids, nonsteroidal anti-inflammatory drugs and antihistamines can relieve symptoms in a small number of patients. New biologics directly targeting IL-1-related inflammatory pathways have shown good efficacy, and targeted therapy has further deepened our understanding of the molecular mechanism of NLRP12-AID. The mechanism of anakinra resistance is still unclear, and imbalances in or compensation by other signaling pathways may be present. However, the disease can be subdivided according to its different responses to IL-1 inhibitors, and in-depth study of the underlying mechanism of its pathogenesis and progression may facilitate the development of more direct and effective targeted drugs.

The rarity of the disease has limited studies related to the pathogenesis and treatment protocols. The published studies are case reports of a small number of patients, and most tend to disclose new mutations and positive results. Therefore, long-term effectiveness and safety data are lacking for emerging drugs for pediatric patients. Although a certain understanding of these rare diseases has been achieved, clinicians are still seeking answers to many questions. Some patients with mutations at the same gene locus have different clinical symptoms, and the role of the natural environment in disease progression is poorly understood. Several emerging drugs can be used for the treatment of pediatric patients, but which drugs will benefit specific patients the most is unknown, and stratified treatment has not yet been realized. Fortunately, animal models of autoinflammatory diseases, advances in genetics, the availability of big data, and the advent of the cloud computing era [53] are highly beneficial to the diagnosis and treatment of such diseases.

## Data Availability

All data generated or analyzed during this study are included in this published article.

## Abbreviations

NLRP12: Nucleotide-binding leucine-rich repeat-containing receptors 12
SAIDs: Systemic autoinflammatory diseases
NLRP12-AID: NLRP12-related autoinflammatory disease
FCAS2: Familial cold autoinflammatory syndrome 2
FMF: Familial Mediterranean fever
TRAPS: TNF receptor-associated periodic syndrome
HIDS: Hyperimmunoglobulin D syndrome
CAPS: Cryopyrin-associated periodic syndrome
NOMID: Neonatal-onset multisystem inflammatory disease
NLRs: Nucleotide-binding domain and leucine-rich repeat-containing receptors
PAMPs: Pathogen-related molecular patterns
DAMPs: Damage associated molecular patterns
LRR: Leucine-rich repeats
IL-1: Interleukin-1
NF-κB: Nuclear factor kappa B
